# Preimplantation Genetic Testing and Embryo Mosaicism: A Critical Narrative Review of Clinical Outcomes and Controversies

**DOI:** 10.1155/ogi/9009681

**Published:** 2026-07-21

**Authors:** Daichi Inoue, Yoshimasa Asada

**Affiliations:** ^1^ Department of Reproductive Medicine, Asada Ladies Nagoya Clinic, Nagoya, Aichi, Japan

**Keywords:** aneuploidy, chromosomal mosaicism, embryo transfer, preimplantation genetic testing

## Abstract

**Background and Aims:**

Preimplantation genetic testing for aneuploidy (PGT‐A) is widely used to select euploid embryos in patients at higher risk of transmitting genetic abnormalities during assisted reproductive technology (ART). Advances in PGT‐A have resulted in the increased detection of embryo chromosomal mosaicism. This review aims to update clinicians and researchers on the implications of mosaicism in ART and emerging clinical outcomes from the use of mosaic embryo transfer cycles.

**Methods:**

A critical narrative review of the current literature was conducted, focusing on clinical outcomes of mosaic embryo transfers, the reliability of mosaicism detection in blastocyst trophectoderm biopsy, and controversies surrounding mosaicism thresholds and their clinical significance in ART. Literature was identified through searches of PubMed and relevant databases focusing on studies related to PGT‐A and embryo mosaicism. As this is a narrative review, no statistical analysis was performed. We conducted a critical narrative review of articles published between January 2015 and May 2026 identified through searches of PubMed, Embase, and the Cochrane Library using terms related to PGT‐A, mosaicism, and IVF outcomes, including original studies, society guidelines, and position statements.

**Results:**

Recent findings demonstrate that mosaicism detected in blastocyst trophectoderm does not always reflect the chromosomal constitution of subsequent embryonic–fetal development. Mosaic embryo transfers can result in healthy clinical outcomes, avoid embryo wastage, and potentially reduce the number of ART cycles. However, such transfers result in reduced implantation rates and higher miscarriage rates, notably with embryos exhibiting complex and high‐level mosaicism. Genetic testing is more reliable with high‐level mosaicism, and defining a high‐level threshold presents one strategy to prioritize mosaic embryos for transfer.

**Conclusion:**

Future research should determine the clinical factors that may influence mosaicism and whether a threshold of mosaicism can improve clinical outcomes. Understanding the benefits versus potential risks of mosaicism will provide important information for patients, counselors, and clinicians in the selection and management of mosaic embryos in ART.

**Summary**
• What’s already known about this topic?
◦ Advances in PGT‐A have led to increased detection of mosaic embryos in ART.
◦ The benefits and potential risks of mosaic embryo transfers in ART have been reported.
◦ Despite current controversy regarding PGT‐A, studies indicate that high‐level mosaicism detected by PGT‐A results in different clinical outcomes.
• What does this study add?
◦ This review presents a balanced update to clinicians and researchers on the implications of the prenatal detection of embryo mosaicism in ART, covering current controversy around PGT‐A and screening for mosaicism and emerging clinical outcomes from the increasing use of mosaic embryo transfer cycles.

## 1. Introduction

Preimplantation genetic diagnosis has been widely used to screen and select embryos for patients at higher risk of transmitting genetic abnormalities during in vitro fertilization. Advances in genetic analysis have led to preimplantation genetic testing for aneuploidy (PGT‐A) and the preferential selection of euploid embryos for transfer in assisted reproductive technologies (ART), with the goal of increasing the efficiency and clinical outcomes of transfer cycles [1, 2]. Contemporary PGT‐A has resulted in the increased detection of chromosomal mosaicism in embryos (containing a mix of euploid and aneuploid cells). The aim of this narrative review is to update both clinicians and researchers on the implications of mosaicism in ART, the controversy that remains in the application of PGT‐A and screening of mosaic embryos, and the emerging clinical outcomes reported from the increasing use of mosaic embryo transfer cycles.

We searched PubMed, Embase, and the Cochrane Library for articles published between January 2015 and May 2026, using combinations of the terms ‘preimplantation genetic testing,’ ‘PGT‐A,’ ‘mosaicism,’ ‘mosaic embryo,’ ‘aneuploidy,’ and ‘IVF outcome.’ We included original studies, society guidelines, and position statements reporting clinical or laboratory outcomes of mosaic embryos; we excluded non‐English articles, conference abstracts without full data, and studies not reporting reproductive outcomes.

### 1.1. Causes of Mosaic Embryos

Embryonic chromosomal mosaicism is largely the result of different postzygotic mitotic errors, such as anaphase lagging, endoreplication, and nondisjunction, whereas uniform aneuploidy results from meiotic errors [1, 2]. Mosaicism can impact single or multiple chromosomes with copy number variation and can include whole‐chromosome or subchromosomal abnormalities. The subtypes of mosaicism—such as monosomic, trisomic, entire chromosomal, or segmental changes—change in frequency with maternal age, including the reported overall higher frequency of embryonic mosaicism in younger patients, and the increasing complexity of mosaic errors in embryos from patients with advancing age [3].

Distinct cell types of developing blastocysts, including the trophectoderm (TE) and inner cell mass, may be impacted differently by changes in the distribution and/or degree of mosaicism during embryonic development. Such changes include the reported marginalization of aneuploid cells away from the inner cell mass of developing mosaic embryos [4] and the proposed‘ self‐correction’ of mosaicism during embryonic development [5]. Cellular marginalization or self‐correction of mosaicism presents a challenge for typical screening strategies of PGT‐A using TE biopsies or proposed noninvasive cell‐free DNA sampling [6], as screening results based on TE or cell‐free DNA (also potentially confounded by DNA from apoptotic cytotrophoblast cells) samples from earlier blastocyst stages may not reflect the level of mosaicism at later stages of embryonic and fetal development.

Recent studies, notably by Bolton et al. from the Zernicka‐Goetz group, have elucidated the mechanisms of ‘self‐correction’ in mosaic embryos. Their work demonstrates that aneuploid cells in the ICM are preferentially eliminated via apoptosis, whereas aneuploid cells in the TE exhibit reduced proliferation. This lineage‐specific depletion ensures that the fetal lineage maintains chromosomal integrity, a critical consideration when interpreting PGT‐A results [7].

### 1.2. Detection and Screening of Mosaic Embryos and Potential Challenges

#### 1.2.1. Detection and Screening of Mosaic Embryos

Contemporary methods used for PGT‐A, such as array comparative genomic hybridization and advances in next‐generation sequencing (NGS) technology, have increased the breadth and complexity of chromosomal analysis and have resulted in the increased detection of intermediate copy number on chromosomal analysis, indicating embryo chromosomal mosaicism [1, 2]. The reported incidence of mosaicism detected in embryo screening varies widely, from 4% to 44%, between different fertility clinics [1, 2]. Such wide variation in the incidence of mosaicism is likely to reflect the use of different PGT‐A platforms, analytical tools and parameters, different patient age groups, as well as distinct culture conditions and embryology protocols of the different centers [1, 2]. This variation highlights the need for standardized procedures and more detailed studies to more accurately determine the key factors that influence the detection of mosaic embryos by PGT‐A.

Technical limitations and the relatively high misdiagnosis rate of NGS add to the complexity of accurately reporting the true degree of mosaicism in embryos, with a misdiagnosis rate for NGS of approximately 18% reported by Popovic et al. [8]. Adding to this complexity, different fertility centers and testing companies have varying cut‐off thresholds for assigning ‘mosaic’ and for stratifying the degree of mosaicism, such as low‐ or high‐level thresholds. The Preimplantation Genetic Diagnosis International Society (PGDIS) suggests a typical cut‐off value (degree of mosaicism) for the assignment of ‘euploid’ as < 20% and ‘aneuploid’ as > 80%, resulting in a broad 20% to 80% range of mosaicism [9]. As described in further detail below, more recent studies have used a cut‐off threshold of approximately 50% for low‐level versus high‐level assignment.

Different studies have demonstrated poor concordance rates between the level of mosaicism detected in the TE versus the inner cell mass and developing embryo biopsy samples [8, 10, 11]. This poor concordance may reflect inherent biological variation of cell cycle stages and numbers sampled, as well as the loss of aneuploid cells by proposed marginalization and self‐correction of mosaicism during embryonic development. Recent studies have described a higher concordance for high‐level mosaic TE samples and concluded that, despite a low cytogenetic concordance rate caused by chromosomal mosaicism in blastocysts, a single TE biopsy could correctly predict whether the inner cell mass consists of predominantly normal or abnormal cells in most cases [12]. It is possible that high levels of mosaicism may escape pathways that remove aneuploid cells, such as apoptosis, marginalization, or self‐correction processes, during embryonic–fetal development. Together, these findings highlight the challenges of detecting ‘low‐level’ mosaicism, the improved reliability of using a higher threshold for assignment of mosaicism (e.g., 50%), as well as the need to further understand the clinical implications of ‘high‐level’ mosaicism in ART.

A central controversy in this field concerns the reliability of the mosaic classification itself. Re‐biopsy studies indicate that a substantial proportion of embryos labelled ‘mosaic’ by NGS‐based PGT‐A are in fact euploid and that predictions based on low‐to‐moderate mosaicism percentages may be of limited clinical value [12, 13]. This raises the possibility that current reporting practices contribute to the disposal of embryos with normal reproductive potential. These limitations, together with the retrospective design and modest sample sizes of most outcome studies, temper the strength of any recommendation.

#### 1.2.2. Clinical Factors Impacting Mosaic Embryos in ART

Recent studies have expanded our understanding of patient‐related and clinical protocol factors in ART that may impact the degree and types of mosaicism detected by PGT‐A, as well as the challenges in defining factors (e.g., chromosomal characteristics) that affect mosaicism. Distinct PGT‐A‐derived mosaic embryo profiles have been described and may be associated with different clinical conditions and indicators, such as the higher mosaicism associated with younger or advancing maternal age and with lower morphology grade embryos and vitrification [3, 14, 15]. Such indicators may have significant implications for patient inclusion criteria in studies of embryo mosaicism.

Investigations from different groups have indicated that the clinical protocols used in ART, such as those requiring consideration of male factor infertility and associated fertilization methods, can impact the degree of embryo mosaicism. For instance, a higher degree of embryo mosaicism has been described for male factor infertility compared with nonmale factor infertility patients [16]. A more recent study reported that the incidence of mosaicism was increased in embryos derived from abnormal compared with normal sperm, and the investigators proposed that sperm quality was an independent factor associated with embryo mosaicism in ART [17]. Therefore, more studies may be warranted to evaluate the characteristics of male factor infertility or abnormal sperm associated with mosaicism to determine the infertile couples for whom PGT‐A would be most beneficial.

Different studies of the incidence of mosaicism and its correlation with specific chromosome characteristics have produced conflicting findings, and many past studies of chromosomes involved in aneuploidy used early screening methods, such as fluorescent in situ hybridization, which lack the breadth and detail of current NGS platforms. However, recent studies using current PGT‐A platforms have also described different findings for the relationship between specific chromosomes and the prevalence of mosaicism in blastocytes. For example, one study investigating chromosome length and the detection of mosaicism in blastocysts reported a positive correlation between mosaicism and chromosome length [18], contrasting with another study that reported a higher incidence of mosaicism with shorter chromosomes [15], adding to the overall lack of consensus regarding chromosomes susceptible to mosaicism.

## 2. Clinical Outcomes of Mosaic Embryo Transfer in ART

With more details of clinical outcomes emerging from the increased application of mosaic embryos, it is important to understand the complexity and controversy surrounding the screening, use and/or exclusion of mosaic embryos in ART.

### 2.1. PGT‐A in ART: Controversy Remains

Some investigators have presented the view that PGT‐A should not be used in ART, with the rationale mostly based on selected randomized control trials. They conclude that PGT‐A negatively impacts live birth rates and provides no clear benefits to patients and propose that the self‐correction from the blastocyst stage eliminates any role for embryo biopsy [19]. However, this position is derived largely from an opinion‐based synthesis of a selected set of trials rather than from new primary data, and it equates the absence of an improvement in cumulative live birth rates with an absence of any clinical benefit, without accounting for per‐transfer efficiency, reductions in miscarriage, or shortened time to live birth. Other investigators have described key flaws in the experimental design of the associated randomized control trials, such as the strategy of embryo randomization, selection of good prognosis and young patients, as well as practical limitations in the choice of reported outcomes, such as cumulative live birth rates [20]. Such limitations in these trials may confound conclusions and restrict the generalizability of findings to wider patient groups. This critique is itself a methodological commentary that reinterprets existing trials rather than providing independent empirical evidence, so its arguments, although compelling, remain to be confirmed in prospectively designed studies. A recent review noted that PGT‐A, as a selection tool, was not designed to improve cumulative live birth rates, as it cannot increase the pool of embryos available for selection from a given cycle [21]. This review also reported that PGT‐A did not impact the cumulative live birth rates when all age groups were considered and proposed that the ‘nonselection’ of mosaic embryos in the transfer pool may explain small differences reported in some studies. As a narrative review, this analysis is nonetheless subject to selection and interpretation bias, and its central claim rests on a conceptual argument about the intended purpose of PGT‐A rather than on a pooled reanalysis of primary outcome data.

Numerous studies have reported improved outcomes from PGT‐A‐selected embryos (many reviewed by Viotti [22] and Seckin and Forman [21]). Recent reports have described the improved ongoing pregnancy rates from NGS versus array comparative genomic hybridization screening [23] and improved live birth rates—particularly in older patients—as a result of the use of PGT‐A‐selected embryos [24]. Because such observational findings are based on patients who reached embryo transfer, they are susceptible to selection bias, whereby better‐prognosis patients with available euploid embryos are preferentially represented. This comparison was drawn from retrospective data in which the two platforms were applied over different periods, so the apparent improvement may partly reflect concurrent advances in laboratory and embryology practice rather than the screening platform alone. Such findings are consistent with an earlier review and meta‐analysis that described increased live birth and ongoing pregnancy rates for PGT‐A versus control groups of women aged > 35 years, but not for those aged ≤ 35 years [25]. This meta‐analysis pooled studies that differed in biopsy stage, screening platform, and mosaicism definitions and reported outcomes on a per‐transfer rather than per‐cycle basis; the resulting heterogeneity and the subgroup nature of the age‐stratified findings warrant cautious interpretation. Therefore, patient age groups and the types and potential selection bias of embryo transfers (including the nonselection of mosaic embryos) require careful consideration for investigations of PGT‐A [21, 22].

### 2.2. Positive Outcomes From Mosaic Embryos in ART

There are increasing numbers of reports describing euploid pregnancies resulting from the transfer of PGT‐A‐defined mosaic embryos [22, 26–28]. A key benefit of using mosaic embryos is the avoidance of embryo wastage, especially for patients with limited embryos available for ART. A summary of reported clinical outcomes from mosaic embryo transfer is provided in Table 1. To date, the potential limitations of studies using PGT‐A‐defined mosaic embryos include the relatively small numbers of patients, retrospective analysis, inclusion criteria, and the age groups examined, so caution is required for extrapolation to all mosaic embryos and patient groups. Many of these studies also lack complete information regarding follow‐up results in prenatal and postnatal periods, including results of genetics tests (such as amniocentesis or postnatal karyotypes). However, the reports of PGT‐A‐assigned mosaic embryos in ART appear to have generally resulted in babies with a normal karyotype when analyzed. There are some exceptions to positive outcomes with mosaic embryo transfers, and a number of clinical outcomes in ART may be negatively impacted by mosaic embryo transfer, as detailed below.

**TABLE 1:** Clinical outcomes from mosaic embryo transfer clinical outcomes from preimplantation genetic testing‐aneuploidy assigned mosaic embryos used in ART, highlighting the different clinical outcomes between low‐level and high‐level mosaic embryo transfer, as well as complex mosaic embryo transfer. All mosaic embryos were defined by next‐generation sequencing and array genomic comparison hybridization.

Ref.	Mosaicism (%^a^)	Embryos (cycles)	IR/OIR (%)	MR (%)	PR/OPR (%)	LBR (%)	Pre/neo^b^
	Low‐level versus high‐level mosaicism:						
Munné et al. [29]	< 20	1045	70/63	10	ND	ND	ND
	20–80	143	53/40	25	ND	ND	
	20–40, Cx	17	12/12	100	0	0	
	> 40–80, Cx	4	0/0	—	—	—	

Lin et al. [30]	> 20–< 50	83 (83)	52/‐	5	47/47	45	Normal
	50–80	25 (25)	52/‐	31	52/36	36	Normal

Munné et al. [23]	< 20	1338	83/77	7	92/‐	ND	Normal^c^
	20–40	105	63/50	16	ND		
	20–80	253	49/37	25	49/‐		
	> 40–80	48	40/27	32	ND		
	20–80, Cx	35	17/9	50	ND		

Spinella et al. [27]	< 20	251 (250)	55/‐	8	‐/46	47	Normal^d^
	20–80	78 (77)	38/‐	8	‐/30	31	
	< 50	45 (44)	49/‐	7	‐/41	42	
	≥ 50	33 (33)	24/‐	9	‐/15	15	

*Note:* Cx, complex mosaicism; Pre/Neo, prenatal/neonatal analysis.

Abbreviations: ART, assisted reproductive technologies; IR, implantation rate; LBR, live birth rate; MR, miscarriage rate; ND, no data; OIR, ongoing implantation rate; OPR, ongoing pregnancy rate; PR, pregnancy rate.

^a^For preimplantation genetic testing of aneuploidy, euploid is typically defined as < 20% mosaicism.

^b^Karyotype/fluorescent in situ hybridization analysis of amniotic fluid, chorionic villus and/or fetal/baby tissue samples.

^c^Only 31% of ongoing pregnancies were examined.

^d^All ongoing pregnancies were examined.

### 2.3. Negative Impacts of High‐Level Mosaic Embryo Transfer in ART

Several studies have presented negative clinical outcomes from mosaic embryo transfers, such as reduced implantation rates [29], reduced live birth rates [31], and higher miscarriage rates [2, 29]. Investigations into the impact of the degree of embryo mosaicism have described higher miscarriage rates [30] and reduced ongoing pregnancy rates [23] with high‐level compared with low‐level mosaic embryo transfers Table 1. The reported association between mosaic embryo transfer and miscarriage should be interpreted with caution. A baseline miscarriage rate of approximately 15% is observed in natural conception, partly attributable to undetected (mosaic) aneuploidy. Outcomes after mosaic embryo transfer are highly heterogeneous and depend on the degree and type of mosaicism: while pooled registry data report lower implantation (46.7% vs. 56.4%) and live‐birth rates (36.8% vs. 49.8%) and higher miscarriage (24.7% vs. 9.0%) compared with euploid transfers, low‐level mosaic embryos have shown outcomes comparable to euploid embryos in nonselection studies [12], and Lin et al. [30] reported similar live‐birth rates between low‐ and high‐mosaicism groups. The smaller sample sizes underlying these figures further limit firm conclusions. Therefore, current evidence indicates that it would be beneficial to select low‐level mosaic embryos for transfer cycles, although such selection may not be possible for patients with few transfer‐grade embryos. The clinical relevance of embryo mosaicism may be important for older patients with increasing levels of ‘complex’ mosaicism, based on its more significant correlation with poor embryonic development potential and pregnancy outcomes compared with other types of mosaicism, such as single, double, or segmental mosaic embryos [22, 29]. Table 1 summarizes the clinical outcomes from mosaic embryo transfer.

A recent report also showed that low‐level embryonic mosaicism can persist to fetal mosaicism during pregnancy, with adverse outcomes in ART [32]. Adverse clinical outcomes also arise from confined placental mosaicism in ART, such as an increased risk of impaired fetal growth [33]. A feature lacking in many studies of PGT‐A‐assigned embryos is the lack of chromosomal analysis of miscarriage samples, so the karyotype remains unknown for many failed pregnancies resulting from mosaic embryo transfer [23]. Negative outcomes from the use of mosaic embryos require careful consideration in ART, and there are important implications for the ongoing prenatal monitoring of patients receiving mosaic embryos.

## 3. Future Directions

Future prenatal diagnosis in ART will require a balanced consideration of the potential benefits (e.g., using limited embryos) versus emerging risks (e.g., elevated adverse pregnancy outcomes) of using mosaic embryos. Further research is required to address gaps in understanding the potential implications of different subtypes and mosaicism loads on the prioritization, management, and subsequent monitoring of mosaic embryo transfers in ART.

### 3.1. Controversy in the Management of Mosaic Embryos in ART

The recent PGDIS position statement on the use of mosaic embryos in ART recommended reporting the apparent % mosaicism and chromosome abnormalities identified in embryos, as well as refraining from classifying mosaic embryos as unsuitable for transfer [9]. A subsequent review of the PGDIS position statement by the ‘International Do No Harm Group in IVF’ presented opposing views in the field, reflecting those of the investigators mentioned above who propose that no PGT‐A is warranted in ART [34]. With a balanced consideration of the above findings, the clinical application of PGT‐A and detection of mosaicism should consider different patient groups and infertility indicators (e.g., younger versus older groups, prior history of failure of pregnancy/ART, and male factor infertility) and the importance of the degree (particularly high level) and type of mosaicism (e.g., complex mosaicism in older patients). Such considerations will provide valuable information for many aspects of ART, such as genetic counselling and decision‐making, clinical management, and ongoing prenatal evaluation.

### 3.2. Standardized Protocols for Prioritization of Mosaic Embryos in ART

Different studies have described the potential use of standardized criteria and clinical factors that may be applied to optimize outcomes with mosaic embryos. For instance, there have been reports suggesting that the thresholds of mosaic load (e.g., 50%) [13] may be used as a guideline to balance false positive/negative screening results and ultimately avoid embryo wastage versus adverse pregnancy outcomes from incorrect assignment of embryo ploidy or the use of complex and high‐level mosaic embryos. Recent research has presented the potential for other factors and biological targets to complement PGT for screening of mosaic embryos, such as mitochondrial DNA content, which has been reported to be higher in PGT‐A‐defined mosaic versus euploid embryos, with mosaic embryos with lower mitochondrial DNA content improving pregnancy outcomes [35]. Other studies have targeted the future clinical potential of noninvasive PGT using spent culture media (cell‐free DNA) for screening mosaic blastocysts. One study described an increased concordance between media and TE biopsy with higher‐level mosaicism and the recommendation of longer culture prior to sampling for noninvasive PGT [36]. Another study reported poor correlation between cell‐free DNA and TE biopsy results, as well as high rates of DNA amplification failure with noninvasive PGT [37]. The clinical relevance of noninvasive screening approaches will require further research, as well as careful consideration of changing patterns of mosaicism during embryonic–fetal development and age. Integrating the above evidence, the decision to transfer a mosaic embryo should be individualized and made only after euploid embryos, where available, have been prioritized. When no euploid embryo is available, a stepwise prioritization framework can guide selection (Table 2 [9, 38]): low‐level mosaicism is generally preferred over high‐level mosaicism, monosomic over trisomic involvement, and single or segmental changes over complex mosaicism, reflecting their respective associations with implantation, miscarriage, and the potential for adverse fetal outcomes. The number of chromosomes involved and the specific chromosomes affected—particularly those compatible with viable aneuploidy syndromes or uniparental disomy—should additionally inform prioritization. Critically, this framework is intended to support, rather than replace, comprehensive genetic counselling, and the transfer of any mosaic embryo should be accompanied by a clear discussion of the residual uncertainty of TE‐based prediction [39] and the recommendation for prenatal diagnostic confirmation. In this way, mosaic embryos can be used to avoid embryo wastage and reduce additional ART cycles while limiting the risk of adverse pregnancy and neonatal outcomes.

**TABLE 2:** Prioritization framework for the transfer of mosaic embryos. Suggested stepwise criteria for prioritizing preimplantation genetic testing for aneuploidy (PGT‐A)‐defined mosaic embryos for transfer when no euploid embryo is available, together with the corresponding rationale. Prioritization should follow comprehensive genetic counselling and be confirmed by prenatal diagnosis.

Parameter	Higher priority for transfer	Lower priority for transfer	Rationale
Mosaicism level	Low‐level (e.g., < 50%)	High‐level (e.g., ≥ 50%)	Higher level associated with reduced implantation and higher miscarriage rates
Chromosome copy change	Monosomy	Trisomy	Lower risk of viable aneuploidy at birth
Extent of involvement	Single chromosome/segmental	Complex (multiple chromosomes)	Complex mosaicism correlates with poorer development and outcomes
Chromosome identity	Chromosomes without viable aneuploidy or UPD risk	Chromosomes compatible with viable aneuploidy syndromes or UPD	Reduces risk of an affected liveborn child
Patient context	Limited or no euploid embryos	Euploid embryo available	Avoids embryo wastage while preserving a safety margin
Prerequisite for all transfers	Genetic counselling + planned prenatal diagnostic confirmation	—	Mitigates residual uncertainty of TE‐based prediction

*Note:* PGT‐A, preimplantation genetic testing for aneuploidy; TE, trophectoderm; UPD, uniparental disomy.

## 4. Conclusions

There is an increasing understanding that mosaicism detected in TE biopsies may not reflect the chromosomal constitution of whole embryos and subsequent fetal development. Numerous reports show that the use of ‘putative’ mosaic embryos, in the absence of more suitable embryos, can result in healthy clinical outcomes in ART. The clinical advantages of mosaic embryo transfer include the avoidance of embryo wastage and potential reduction in ART cycles required for patients with limited embryos. While the application of PGT‐A and selection or nonselection of mosaic embryos remain controversial, recent research indicates there may be potential benefits from detailed PGT‐A‐based detection of mosaic embryos during ART, such as avoiding the reduced implantation rates and higher miscarriage rates reported for mosaic embryo transfer, particularly for high‐level mosaicism. In future applications, rather than reserving the management of mosaic results for specific subgroups, an individualized approach is warranted. This is particularly relevant for women of advanced age, who produce fewer embryos and have a higher probability of mosaic findings; a restrictive policy in this group risks the disposal of embryos with genuine reproductive potential.

Overall, more research is required to carefully define the clinical factors that influence the degree of mosaicism and to determine whether copy‐number thresholds or degree of specific types of mosaicism can accurately predict mosaicism in later embryonic–fetal development and improve clinical outcomes. Defining high‐level mosaicism, which may also provide increased reliability for embryo screening, presents one strategy for prioritizing mosaic embryos for transfer. Future research should aim to determine the potential risks associated with specific types of mosaicism (e.g., complex mosaicism and specific chromosomes), with the overall goal to provide evidence‐based information for patients, counselors, and clinicians in the detection, selection, and management—including any required prenatal and postnatal follow‐up—of mosaic embryos in ART.

## Author Contributions

The authors conceived and designed the manuscript, conducted the literature review, and wrote the entire article. Daichi Inoue had full access to all of the data in this study and takes complete responsibility for the integrity of the data and the accuracy of the data analysis.

## Disclosure

All authors have read and approved the final version of the manuscript. The funding sources had no role in study design, data collection, analysis, interpretation, or manuscript preparation.

## Ethics Statement

The authors have nothing to report.

## Consent

The authors have nothing to report.

## Conflicts of Interest

The authors declare no conflicts of interest.

## Data Availability

Data sharing is not applicable to this article as no datasets were generated or analyzed during the current study.
